# Cardiac biomarkers for risk stratification in newly diagnosed high-risk multiple myeloma in the GMMG-CONCEPT trial

**DOI:** 10.1186/s40959-025-00358-x

**Published:** 2025-07-03

**Authors:** Lisa B. Leypoldt, Linlin Guo, Britta Besemer, Mathias Hänel, Marc-S. Raab, Christoph Mann, Christian S. Michel, Hans Christian Reinhardt, Igor Wolfgang Blau, Martin Görner, Yon-Dschun Ko, Maike de Wit, Hans Salwender, Christof Scheid, Ullrich Graeven, Rudolf Peceny, Peter Staib, Annette Dieing, Hartmut Goldschmidt, Carsten Bokemeyer, Tanja Zeller, Dirk Westermann, Katja C. Weisel, Raphael Twerenbold, Antonia Beitzen-Heineke

**Affiliations:** 1 Department of Hematology, Oncology and Bone Marrow Transplantation with Section of Pneumology, University Medical Center Hamburg-Eppendorf, Hamburg, Germany; 2 Department of Cardiology, University Medical Center Hamburg-Eppendorf, University Heart & Vascular Centre Hamburg and University Center of Cardiovascular Science, Hamburg, Germany; 3 Department of Hematology, Oncology, Immunology, Rheumatology and Pulmonology, University Hospital of Tuebingen, Tuebingen, Germany; 4 Department of Hematology, Oncology and Bone Marrow Transplantation, Klinikum Chemnitz, Chemnitz, Germany; 5 Internal Medicine V, Hematology, Oncology and Rheumatology, GMMG Study Group, Heidelberg University Hospital, Heidelberg, Germany; 6 Department of Hematology, Oncology and Immunology, University Hospital of Gießen and Marburg, Marburg, Germany; 7 Department of Internal Medicine III, University Medical Center Mainz, Mainz, Germany; 8 Department of Hematology and Stem Cell Transplantation, University Hospital Essen, University Duisburg-Essen, German Cancer Consortium (DKTK Partner Site Essen), Essen, Germany; 9 Department of Hematology and Oncology, Charité – Universitätsmedizin Berlin, corporate member of Freie Universität Berlin and Humboldt-Universität Zu Berlin, Berlin, Germany; 10 Department of Hematology, Oncology and Palliative Care, Klinikum Bielefeld Mitte, Bielefeld, Germany; 11 Department of Internal Medicine, Hematology and Oncology, Johanniter Krankenhaus Bonn, Bonn, Germany; 12 Department of Internal Medicine, Hematology and Oncology, Vivantes Klinikum Neukölln, Berlin, Germany; 13 Asklepios Tumorzentrum Hamburg, AK Altona and AK St. Georg, Hamburg, Germany; 14 Department of Internal Medicine I, University Hospital Cologne, Cologne, Germany; 15 Department of Hematology, Oncology and Gastroenterology, Maria Hilf Kliniken, Mönchengladbach, Germany; 16 Department of Oncology, Hematology and Stem Cell Transplantation, Klinikum Osnabrück, Osnabrück, Germany; 17 Department of Hematology and Oncology, St. Antonius Hospital Eschweiler, Eschweiler, Germany; 18 Department of Hematology and Oncology, Vivantes Klinikum Am Urban, Berlin, Germany; 19 German Center for Cardiovascular Research, Partner Site Hamburg/Lübeck/Kiel, Hamburg, Germany; 20 Institute for Cardiogenetics, University Hospital Schleswig-Holstein, University of Lübeck, Lübeck, Germany; 21 Department of Cardiology and Angiology, University Heart Center Freiburg-Bad Krozingen, Faculty of Medicine, University of Freiburg, Freiburg, Germany

## Abstract

Cardiovascular adverse events (CVAE) are clinically relevant side effects during treatment with the proteasome inhibitor carfilzomib. We investigated the predictive value of cardiac biomarkers for onset of CVAE in patients with newly diagnosed high-risk multiple myeloma treated with isatuximab, carfilzomib, lenalidomide, and dexamethasone in the GMMG-CONCEPT study (NCT03104842).

Patients included in this prospective, multicenter correlative study were eligible if a serum sample before treatment initiation and at ≥ 1 later study time point were available. N-terminal pro-b-type natriuretic peptide (NT-proBNP) and high-sensitive Troponin I (hsTropI) were measured using immunoassays. Time-to-event analyses were performed using Kaplan–Meier estimators and log-rank test was used for statistical analysis. Among 126 patients included in this study, 40 reported incident CVAE. No significant differences were observed for age, sex, cardiovascular risk factors and cardiovascular comorbidities between patients who experienced CVAE compared to patients without CVAE. NT-proBNP levels were elevated at baseline in 96 (76%) patients. Neither baseline levels nor change in NT-proBNP level during early induction cycles were predictive for the occurrence of CVAE. In contrast, elevation of hsTropI above the 99th percentile was rare. Patients with hsTropI level ≥ 2.9 ng/L, corresponding to the lower limit of quantification, showed a higher risk for CVAE compared to patients with hsTropI < 2.9 ng/L at baseline (*p* = 0.0023).

In conclusion, in patients with newly diagnosed high-risk multiple myeloma undergoing carfilzomib-based quadruplet treatment, low hsTropI pretreatment levels are of high negative predictive value for the occurrence of CVAE whereas elevated NT-proBNP levels are very common before treatment initiation.

**Graphical Abstract:**

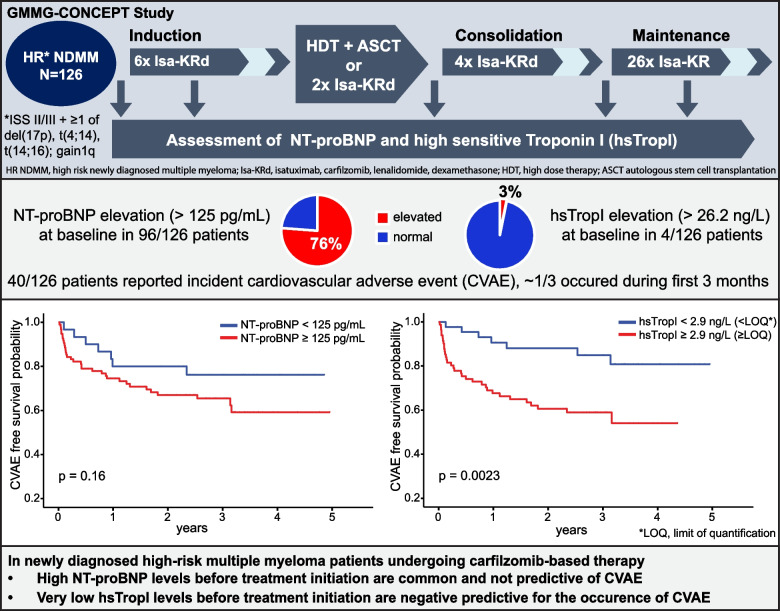

**Supplementary Information:**

The online version contains supplementary material available at 10.1186/s40959-025-00358-x.

## Key Points

• Marked elevation of NT-proBNP levels is common in newly diagnosed multiple myeloma and not predictive of cardiovascular adverse events.

• Low hsTroponin I levels are of negative predictive value for the risk of cardiovascular adverse events in newly diagnosed multiple myeloma.

**Supplementary Information:**

The online version contains supplementary material available at 10.1186/s40959-025-00358-x.

## Introduction

Multiple myeloma (MM) treatment has evolved immensely over the past two decades with monoclonal CD38-directed antibodies, immunomodulatory agents, and proteasome inhibition, as cornerstones of MM therapy [1, 2]. Carfilzomib is a proteasome inhibitor (PI) of the novel generation that irreversibly inhibits the 20S proteasome and has proven its efficacy in combination with standard of care regimens [3–9]. It is approved for relapsed disease after at least one prior line of treatment. While the tolerability of carfilzomib is overall good, cardiovascular side effects, including arterial hypertension, heart failure and arrhythmias, have been described at rates ranging from 5–55% [10–14]. Moreover, risk for thromboembolic events is particularly high in patients treated with combinations of immune-modulating drugs (IMiD) and is further elevated during combination treatment with IMiD and PI [9].

In 2019, the European Myeloma Network together with the Italian Society of Arterial Hypertension considered cardiac biomarkers as not essential parameters in clinical practice for the early detection of cardiotoxicity related to carfilzomib but stated their potential in stratifying patients at risk to develop cardiovascular adverse events (CVAE) [15]. Based on results of retrospective analyses and a prospective study showing that in MM patients with relapsed or refractory (r/r) disease an elevated baseline natriuretic protein (BNP) was predictive of CVAE during carfilzomib-based treatment [16], the European Society of Cardiology (ESC) and International Cardio-Oncology Society developed a risk score for MM patients scheduled to receive treatment with PI or immunomodulatory agents [17], although admitting the lack of prospective data. Besides demographic and cardiovascular risk factors (e.g. age, family history, comorbidities), this risk score also incorporates cardiac biomarkers, namely baseline b-type natriuretic peptide (BNP) or N-terminal (NT)-proBNP and cardiac troponin. The 2022 ESC guideline on cardio-oncology recommends routine measurement of natriuretic peptide prior to PI in high- and very high-risk patients whereas measurement should be considered in low- and moderate-risk patients [18]. However, to date, these recommendations are based on one study in relapsed or refractory MM (RRMM) while prospective studies evaluating the predictive value of these cardiac biomarkers in MM patients with newly diagnosed (ND) disease are missing [16].

Here, we prospectively evaluated the clinical utility of the two cardiac biomarkers NT-proBNP and high-sensitive troponin I (hsTropI) to predict incident CVAE in a cohort of high-risk NDMM patients from the GMMG-CONCEPT trial undergoing carfilzomib-based quadruplet treatment with isatuximab, carfilzomib, lenalidomide, and dexamethasone (Isa-KRd).

## Methods

### Patients and trial design

**Fig. 1:**
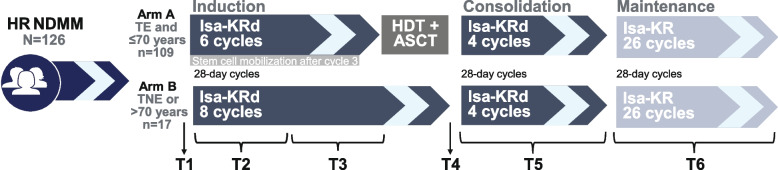
GMMG-CONCEPT study overview. In the GMMG-CONCEPT study, newly diagnosed high-risk multiple myeloma patients were treated with a carfilzomib-based quadruplet therapy, consisting of 6 induction cycles, stem cell mobilization and high-dose melphalan followed by autologous stem cell transplantation for transplant-eligible patients or 8 induction cycles for transplant-noneligible patients. Following, all patients received 4 cycles of consolidation and 26 cycles of maintenance therapy. Serum samples were collected of 126 patients before treatment initiation (T1), during the first 3 induction cycles (T2), during induction cycles 3–6 (8) (T3), before consolidation, during consolidation cycles (T5), and during maintenance cycles (T6). d, dexamethasone; HDT + ASCT, high-dose melphalan treatment followed by autologous stem cell transplantation; HR, high-risk; Isa, isatuximab; K, carfilzomib; NDMM, newly diagnosed multiple myeloma; R, lenalidomide; TE, transplant-eligible; TNE, transplant-noneligible

The GMMG-CONCEPT trial is an academic, investigator-initiated, multicenter phase II trial for high-risk NDMM patients with two treatment arms according to age and eligibility for high-dose treatment (HDT). Exclusion criteria included heart failure New York Heart Association class 3 or 4, uncontrolled arrhythmia, and systemic light chain amyloidosis. All patients receive six cycles of Isa-KRd induction, 4 cycles Isa-KRd consolidation, and 26 cycles of Isa-KR maintenance; transplant-noneligible (TNE) patients receive 2 additional Isa-KRd cycles after induction instead of HDT (Fig. 1).

Detailed treatment procedures have been described previously [19, 20] and are listed in Supplementary Table 1; in brief, treatment cycles lasted 28 days with carfilzomib 36 mg/m^2^ given intravenously (IV) on 6 days per cycle in induction and consolidation, and carfilzomib 70 mg/m^2^ given IV on 2 days per cycle throughout maintenance. The study was conducted according to the Declaration of Helsinki; all patients provided written informed consent and competent authorities and ethics committees approved the trial as well as this correlative study.

### Sample collection and clinical data

Serum samples were collected at various time points throughout the trial: at baseline (T1), during induction cycles 1–3 (T2), during induction cycles 4–6 (4–8 for TNE patients) (T3), after intensification (T4), during consolidation (T5), and during maintenance (T6) (Fig. 1), and were stored at −80°C in a central, dedicated laboratory until analysis. Patients were included in this analysis if serum samples at baseline and at least one later time point were available; hence, the patient population in this study is different from the overall or interim analysis population reported on earlier [19, 20]. Cardiac biomarkers were measured collectively using immunoassays for this post-hoc analysis. NT-proBNP levels were measured using the ARCHITECT NT-proBNP assay (Abbott Diagnostics, Delkenheim, Germany) with a limit of quantification (LOQ) of 8.2 pg/mL. As suggested by current clinical ESC guidelines, NT-proBNP levels above 125 pg/mL were considered elevated [21]. HsTropI was assessed using the Abbott STAT Architect high sensitive troponin I assay (Abbott Diagnostics, Delkenheim, Germany) with a limit of quantification of 1.5–2.9 ng/L and a uniform 99th percentile of 26.2 ng/L. HsTropI levels above the 99th percentile were considered elevated.

Clinical data regarding baseline demographics (including preexisting cardiovascular comorbidities [CVM] and risk factors [CVRF]), treatment course, and incidence of CVAE were assessed via electronic case report forms. CVRF were defined as diabetes mellitus, active smoking status, body mass index [BMI] > 30 kg/m^2^, and dyslipidemia. CVM included arterial hypertension, coronary artery disease, heart failure, left-ventricular diastolic dysfunction, arrhythmia, renal function impairment, and other (Supplementary Table 2). CVAE included arterial hypertension CTCAE grade 3 or 4, myocardial infarction, stroke, venous thromboembolic events, atrial fibrillation, ventricular tachycardia, reduction of left ventricular ejection fraction (LVEF), heart failure, and diastolic dysfunction. Worsening of symptoms and new onset events that were deemed related to study medication by the trial site investigators were considered for this analysis. CVAEs were graded according to Common Terminology Criteria for Adverse Events (CTCAE, version 5.0). Data cut-off for this analysis was on August 1, 2022.

### Statistical analysis

Statistical analyses were performed and figures were generated by using R software (R version 4.2.2). Continuous values are presented as median with interquartile range (IQR), categorical variables are expressed as numbers and proportions (%). Kruskal–Wallis test was used for statistical analysis of continuous variables and chi-squared test for binary variables. The survival function was estimated with Kaplan–Meier estimators and log-rank test was used for statistical analysis. Time-to-event analysis and cox regression were performed with the R package “survival”. *P*-values < 0.05 were considered statistically significant. No adjustment for multiple testing was applied.

## Results

### Patient characteristics

In total, 126 patients were included in this analysis with 2–11 serum samples per patient. Median age of patients was 60 years (range, 35–82 years), 62 patients (49%) were male. Overall, 49 patients (38.9%) presented with at least one CVRF and 64 patients (50.8%) presented with preexisting CVM. The most common cardiovascular risk factor was smoking (*n* = 26, 20.6%), followed by obesity (*n* = 17, 13.5%). The most common preexisting CVM was arterial hypertension (*n* = 51, 40.5%). 87 patients underwent HDT while 39 patients did not. Median duration of treatment was 36 months (IQR, 29, 43 months) with a median dose intensity of carfilzomib of 99.4% (IQR, 79.3–99.9%). Median follow-up time was 3 years (IQR, 2.77–3.23). Detailed patient characteristics can be found in Table 1.

**Table 1:** Patient characteristics at study enrollment

	**All patients** **(*n*= 126)**	**Patients without CVAE** **(*n* = 86)**	**Patients with CVAE** **(*n* = 40)**	***p*-value**
Age (years), median (IQR)	60 (56, 68)	60 (56, 67)	63 (58, 68)	0.16
Male, no. (%)	62 (49)	47 (55)	15 (38)	0.11
BMI (kg/m^2^), median (IQR)	25.7 (23.6, 28.7)	25.7 (23.7, 28.7)	26.2 (23.1, 28.1)	0.86
**Cardiovascular Risk Factors, no. (%)**	49 (38.9)	33 (38.4)	16 (40)	1.00
Active smoking	26 (20.6)	18 (20.9)	8 (20.)	1.00
BMI > 30 kg/m^2^	17 (13.5)	10 (11.6)	7 (17.5)	0.54
Diabetes	13 (10.3)	9 (10.5)	4 (10)	1.00
Dyslipidemia	6 (4.8)	4 (4.7)	2 (5.0)	1.00
**Cardiovascular Comorbidities, no. (%)**	64 (50.8)	39 (45.3)	25 (62.5)	0.11
Arterial hypertension	51 (40.5)	30 (34.9)	21 (52.5)	0.093
Atrial fibrillation	7 (5.6)	5 (5.8)	2 (5)	1.00
Coronary artery disease	7 (5.6)	5 (5.8)	2 (5)	1.00
Heart failure	3 (2.4)	2 (2.3)	1 (2.5)	0.12
Diastolic dysfunction	2 (1.6)	-	2 (5)	0.19
Renal injury	14 (11.1)	11 (12.8)	3 (7.5)	0.57
Other CVM	20 (15.9)	9 (10.5)	11 (27.5)	**0.03**
**Treatment**				
High dose chemotherapy, no. (%)	87 (69)	57 (66)	30 (75)	0.32
Time on treatment (months), median (IQR)	36 (29, 43)	35 (28, 41)	41 (32, 45)	**0.017**
Carfilzomib median dose intensity, median (IQR)	1.0 (0.8, 1.0)	1.0 (0.98, 1.0)	0.96 (0.52, 1.0)	**0.0014**

Kruskal–Wallis test was applied for continuous variables and chi-squared test for binary variables

*BMI* Body mass index, *CVAE* cardiovascular adverse event, *CVM* cardiovascular comorbidity, *IQR* interquartile range, *no.* number

Bold *p*-values indicate significance (*p*-value < 0.05)

### Occurrence of cardiovascular adverse events

Overall, 40 (31.7%) patients were reported to have experienced any CVAE throughout the treatment period. Fourteen patients were reported to have experienced more than one CVAE with 60 CVAE reported in total. Patients who experienced CVAE showed a trend towards a higher prevalence of preexisting arterial hypertension compared to patients who did not experience a CVAE (53 vs. 35%, *p* = 0.093) whereas no differences were observed in age, sex, cardiovascular risk factors, and other preexisting cardiovascular comorbidities (Table 1). Time on treatment was significantly longer in patients who experienced a CVAE compared to those without a CVAE (41 months [IQR, 37–52] vs. 35 months [IQR, 28–41], *p* = 0.017) and median carfilzomib dose was significantly lower in patients who experienced a CVAE (0.96 [IQR, 0.52–1.0] vs. 1.0 [IQR, 0.98–1.0], *p* = 0.0014) (Table 1).

The most common reported CVAE were arterial hypertension (overall, *n* = 24 [in 19% of patients]; CTCAE grade ≥ 3, *n* = 17, 14%), followed by cardiac dysfunction (*n* = 11, 8.7%), and thromboembolic events (*n* = 9, 7%) (Table 2). Patients with cardiac dysfunction included 6 patients reported with heart failure (5%), 3 patients with diastolic dysfunction (2%) and 2 patients with reduction of LVEF (2%) not fulfilling the definition of heart failure (Table 2). Other cardiovascular events reported included atrial fibrillation, ventricular tachycardia, stroke, and myocardial infarction. None of the CVAE were fatal.

**Table 2:** Cardiovascular adverse events (CTCAE classification)

Event	No. of patients, n (%)		
	**All**	**G1-2**	**G3-4**
Arterial hypertension	24 (19)	7 (6)	17 (14)
Myocardial infarction	1 (1)	-	1 (1)
Atrial fibrillation	4 (3)	3 (2)	1 (1)
Ventricular tachycardia	2 (2)	1 (1)	1 (1)
Heart failure	6 (5)	3 (2)	3 (2)
LVEF reduction	2 (2)	2 (2)	-
Diastolic dysfunction	3 (2)	3 (2)	-
Stroke	3 (2)	2 (2)	1 (1)
Thromboembolic event	9 (7)	8 (6)	1 (1)

Grading of cardiovascular adverse events (new onset and/or worsening) was assessed according to CTCAE V5.0

*G* grade, *LVEF* left ventricular ejection fraction, *No* number

One third of the CVAE (20/60, 33%) occurred during the first 3 months of treatment (identical to the first 3 cycles of induction therapy). Only 9 CVAE (9/60, 15%) were reported during later induction cycles 4 to 6; 10 events (10/60, 17%) occurred during consolidation, and 21 CVAE (21/60, 35%) during maintenance treatment. Among patients who experienced a CVAE, the median time from treatment initiation to occurrence of first CVAE was 152 days (IQR, 40–342 days).

### NT-proBNP is frequently elevated at baseline

As NT-proBNP is an established cardiac biomarker indicating higher ventricular wall stress caused by intravascular volume overload [17, 22], we investigated its levels over the course of Isa-KRd treatment. In case of multiple samples belonging to the same time point, analyses were performed with the highest and the earliest value; since results did not differ significantly, the presented results are derived from the highest value in each time period.

Strikingly, about three quarters of these HR NDMM patients (*n* = 96, 76%) showed elevated levels (> 125 pg/mL) of NT-proBNP at baseline (median 229 pg/mL; IQR, 129–504 pg/mL). NT-proBNP levels dropped during induction and intensification with a subsequent slight rise in consolidation and maintenance (Table 3). A significant inverse correlation of NT-proBNP levels and renal function (glomerular filtration rate) was found (r = −0.43, *p* < 0.001).

**Table 3:** NT-proBNP and hsTropI levels during treatment

Time point	No. of samples	NT-proBNP		hsTropI	
		median (IQR) pg/mL	elevated n (%)	median (IQR), ng/L	elevated n (%)
T1	126	229 (129, 504)	96 (76)	3.7 (2.2, 7.0)	4 (3)
T2	74	153 (72, 396)	42 (57)	3.5 (2.3, 5.3)	0
T3	77	148 (75, 296)	43 (56)	3.0 (2.2, 4.9)	1 (1)
T4	75	110 (66, 200)	26 (35)	3.3 (2.0, 5.1)	0
T5	97	146 (71, 349)	53 (55)	3.3 (2.2, 6.2)	2 (2)
T6	77	155 (88, 288)	51 (66)	4.3 (2.6, 7.9)	4 (5)

For NT-proBNP, concentrations > 125 pg/mL were considered elevated, for hsTropI > 26.2 ng/L

*IQR* interquartile range, *No* number, *NT-proBNP* N-terminal pro-b-type natriuretic peptide, *hsTropI* high sensitive Troponin I

Patients with pre-existing CVM as well as patients who developed a new CVAE showed significantly higher NT-proBNP levels during later treatment courses, particularly during maintenance, but not at baseline or during induction cycles (Table 4). No significant differences in NT-proBNP levels were observed comparing patients with or without CVRF (Supplementary Table 3).

**Table 4:** NT-proBNP levels in pg/mL depending on prevalent CVM and incident CVAE

Time point	CVM			CVAE		
	Absent *n* = 62	Present *n* = 64	*p*-value	Absent *n* = 86	Present *n* = 40	*p*-value
T1	192 (102, 476)	250 (158, 522)	0.091	199 (114, 519)	257 (170, 493)	0.26
T2	132 (72, 221)	212 (83, 649)	0.13	158 (81, 386)	148 (67, 541)	0.80
T3	148 (62, 263)	148 (80, 411)	0.25	145 (75, 226)	190 (72, 321)	0.40
T4	87 (62, 136)	122 (92, 353)	**0.007**	97 (63, 185)	120 (73, 219)	0.25
T5	111 (58, 190)	171 (84, 574)	0.016	144 (57, 279)	159 (91, 359)	0.15
T6	131 (56, 186)	224 (115, 379)	**0.008**	130 (58, 251)	205 (151, 396)	**0.0048**

Medians (interquartile range) are displayed. Kruskal–Wallis test was applied, a *p*-value < 0. 5 was considered significant

*CVAE* cardiovascular adverse event, *CVM* cardiovascular comorbidities

### Elevated levels of NT-proBNP are not predictive of CVAE

**Fig. 2:**
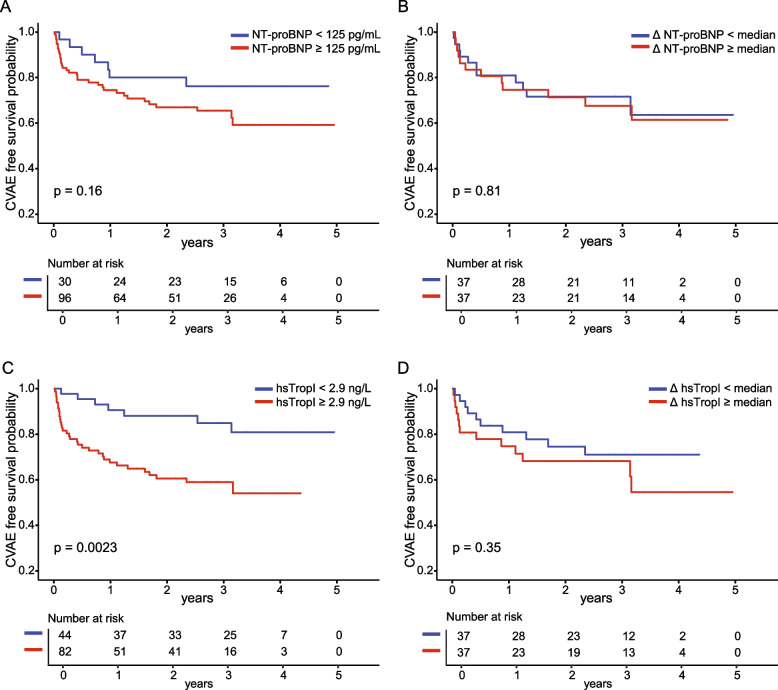
CVAE free survival depending on NT-proBNP and hsTropI levels. NT-proBNP and high sensitive troponin I (hsTropI) levels were measured using immuno-assays in patients before initiation (*n* = 126, T1) and during the first three cycles (*n* = 74, T2) of carfilzomib-based quadruplet therapy for newly diagnosed high risk multiple myeloma within the GMMG-CONCEPT study. Occurrence of cardiovascular adverse events was assessed via electronic case report forms. Time-to-event analysis was performed stratifying patients by (**A**) NT-proBNP levels < 125 pg/mL and ≥ 125 pg/mL (99th percentile), (**B**) by median ΔNT-proBNP (NT-proBNP_T2_-NT-proBNP_T1_), (**C**) by hsTropI levels below or above the lower limit of quantification (LOQ), corresponding to 2.9 ng/L, and (**D**) by median ΔhsTropI (hsTropI_T2_-hsTropI_T1_). Log-rank test was used for statistical analysis. P-values < 0.05 were considered significant

Next, we evaluated a possible relationship between elevated NT-proBNP levels and the occurrence of CVAE during treatment by using time-to-event analyses. Elevated levels of NT-proBNP at baseline were not predictive of new CVAE at any timepoint, neither for elevated vs. normal levels (*p* = 0.16, log-rank test, Fig. 2A) nor for NT-proBNP levels below or above the median (*p* = 0.23, Supplemental Fig. 1A) or using tertiles (*p* = 0.11, Supplemental Fig. 1B).

With absolute NT-proBNP levels not being predictive for the occurrence of CVAE, the highest event rate for CVAE during first treatment cycles, and a previously described value of a natriuretic peptide elevation during cycle one in RRMM [16], we wondered whether an early change in NT-proBNP (ΔNT-proBNP = NT-proBNP_T2 _- NT-proBNP_T1_) could be of value for predicting a CVAE in our patient population. Most patients (48/74) showed a decrease of NT-proBNP levels at T2 compared to T1 (median decrease 143 pg/mL, IQR, -70--483) whereas an increase in NT-proBNP levels was observed in 26/74 patients (median increase 94 pg/mL, IQR, 32.5–398). Time-to-event analysis showed that ΔNT-proBNP levels were not predictive of CVAE when stratified by median (*p* = 0.81, log-rank test, Fig. 2B), again also confirmed when ΔNT-proBNP levels were stratified into tertiles (*p* = 0.58, Supplemental Fig. 1C). Complementary, Cox regression analysis confirmed that baseline NT-proBNP and ΔNT-proBNP were not predictive of CVAE.

### Troponin I is rarely elevated, but has predictive value even within normal ranges

Troponin I is released to the peripheral blood in cases of heart muscle damage and therefore a widely used biomarker of myocardial injury [17, 23, 24]. We thus sought to investigate also hsTropI as potential predictive biomarker for CVAE in this HR NDMM patient cohort. In contrast to NT-proBNP, levels of hsTropI were in general low at baseline (median level of 3.7 ng/L, IQR, 2.2—7.0) and elevated above normal limits (> 26.2 ng/L) in only 4 of 126 patients (3%). After treatment initiation, a total of seven cases with increased levels of hsTropI were found, mostly occurring during maintenance therapy in 4 of 126 patients (3%) (Table 3). Patients with preexisting CVM showed significantly higher levels of hsTropI vs. patients without CVM while patients who developed a new CVAE had significantly higher hsTropI levels only during maintenance (*p* < 0.001) (Table 5). No significant differences in hsTropI levels were observed comparing patients with or without CVRF (Supplementary Table 4).

**Table 5:** HsTropI levels in ng/L depending on prevalent CVM and incident CVAE

Time point	CVM			CVAE		
	Absent *n* = 62	Present *n* = 64	p-value	Absent *n* = 86	Present *n* = 40	*p*-value
T1	3.2 (1.8, 5.4)	4.7 (2.9, 8.8)	**0.0048**	3.5 (2.1, 7.4)	4.2 (3.1, 6.6)	0.15
T2	3.0 (1.9, 4.2)	4.1 (2.3, 7.1)	**0.030**	3.4 (2.3, 4.8)	4.2 (2.2, 8.7)	0.13
T3	2.5 (1.8, 4.0)	4.1 (2.7, 5.6)	**0.0011**	3.2 (2.0, 4.4)	3.0 (2.4, 5.2)	0.74
T4	3.0 (1.8, 4.0)	4.2 (2.9, 6.7)	**0.0025**	3.3 (2.0, 5.4)	3.3 (2.7, 4.5)	0.92
T5	3.0 (1.8, 4.9)	3.8 (2.4, 6.6)	0.051	3.2 (2.1, 6.0)	3.5 (2.2, 6.2)	0.86
T6	3.5 (2.2, 5.5)	5.8 (3.1, 8.8)	**0.016**	3.2 (2.0, 5.4)	6.7 (4.6, 8.3)	** < 0.001**

Medians (interquartile range) are displayed. Kruskal–Wallis test was applied, a *p*-value < 0.05 was considered significant

*CVAE* cardiovascular adverse event, *CVM* cardiovascular comorbidities

To assess the predictive value of hsTropI levels for CVAE, we again performed a time-to-event analysis using different cut-offs. We found that patients with baseline hsTropI levels of ≥ 2.9 ng/L and greater to or equal to the median, respectively, had a significantly higher risk of developing a CVAE during treatment course compared to patients with hsTropI levels < 2.9 ng/L and below the median, respectively (*p* = 0.0023 and *p* = 0.042, respectively, Fig. 2C and Supplemental Fig. 2). When separating patients by median ΔhsTropI (ΔhsTropI [ng/L] = hsTropI _T2_- hsTropI _T1_) (Fig. 2D), no differences in risk of developing a CVAE were observed (*p* = 0.35). In Cox regression analysis, not absolute hsTropI levels at baseline but an early change from baseline (ΔhsTropI [ng/L]) was associated with a trend towards an elevated risk for occurrence of CVAE (hazard ratio 1.11 (1—1.23), *p* = 0.053), even after adjustment for possible confounders (age, sex, preexisting CVM) (hazard ratio 1.1 (1.0—1.2), *p* = 0.090).

### NT-proBNP in patients with more strictly defined CVAE

Since the definition of CVAE was rather broad and to more specifically investigate the carfilzomib-associated CVAE, we performed further analyses using a more strictly defined CVAE (termed CVAE_2_) that excludes stroke and thromboembolic events. In line with the initial CVAE definition, NT-proBNP levels did not differ at baseline or during early treatment cycles but were higher during maintenance in patients who experienced a CVAE_2_ compared to patients who did not experience a CVAE_2_ (Supplemental Table 5). However, when patients were stratified based on the occurrence of cardiac dysfunction, defined as development of heart failure, LVEF reduction and/or diastolic dysfunction, a trend towards higher NT-proBNP levels at baseline was observed in patients who developed cardiac dysfunction compared to patients who did not develop cardiac dysfunction (Supplemental Table 6).

### HsTropI in patients with more strictly defined CVAE

When using the stricter definition of CVAE_2_ that excludes stroke and thromboembolic events, hsTropI levels were higher not only during maintenance but also during induction cycles in patients who experienced a CVAE_2_ (Supplemental Table 6). Similarly, patients with incident cardiac dysfunction showed higher hsTropI levels during later induction cycles (Supplemental Table 7).

## Discussion

Our study is one of the first to prospectively evaluate NT-proBNP and hsTropI as cardiac biomarkers in newly diagnosed MM, in the purely high risk patient population of the multi-center GMMG-CONCEPT trial undergoing Isa-KRd treatment. Cardiovascular side effects were common with rates of 32%, but not leading to treatment discontinuation, similar to what has been reported by others [12–14, 25, 26]. We could show that in this newly diagnosed high risk disease setting, NT-proBNP levels are frequently elevated, however neither the baseline value nor an early change from baseline within the first three induction cycles were predictive for CVAE. In contrast, while hsTropI levels were rarely elevated, levels below the lower limit of quantification at baseline were negative predictive for the occurrence of CVAE.

While the occurrence of CVAE seems relatively high in this cohort compared to what was reported in pivotal trials (ASPIRE, ENDEAVOR, ARROW) [8, 27, 28], it is in line with more recent studies and meta-analyses [12, 25, 29], like Astarita et al. reporting rates of up to 55% in a real-world prospective analysis of Kd/KRd treatment [12] and likely due to heterogeneous definitions of CVAE across studies. In addition, the CTCAE grading used in oncology trials has its shortcomings with regards to reporting on cardiac adverse events [30], and assessment of CVAE in a clinical study that was not primarily designed to investigate cardiovascular events poses a risk of both under- and overreporting CVAE. To address this, analyses in this study were performed using a broad as well as a stricter definition of CVAE.

Reported rates of CVAE did not differ between NDMM and RRMM with an overall approximately 2.3-fold increased relative risk of all-grade heart failure for the use of carfilzomib [26, 31]. It is important to note, however, that the rate of severe CVAE such as stroke and myocardial infarction or other CVAE leading to treatment discontinuation was overall low across trials [14], underlining the frequent but mostly non-severe or well manageable nature of CVAE. In addition, it has been shown that the favorable outcomes associated with carfilzomib treatment, specifically in terms of mitigating progression or mortality, surpass the potential risk of cardiac failure or hypertension for the majority of patients [14], which may be even more important for high-risk MM patients in whom the use of carfilzomib over bortezomib has been shown to be beneficial [32].

It is interesting to speculate about the cause of the here reported commonly elevated NT-proBNP values; severely impaired kidney function could be ruled out both due to inclusion criteria (eGFR ≥ 30 mL/min/1.73 m^2^) and patient characteristics (Table 1), but patients with mild and moderate impairment of kidney function were included and NT-proBNP levels correlated with baseline GFR. Another contributing factor to increased biomarker levels might be increased vascular and circulatory stress and inflammation in these high-risk NDMM patients with high tumor burden where generous hydration e.g. for hypercalcemia may have been needed. However, markedly elevated levels of NT-proBNP have been described in patients with hematologic malignancies independent of heart failure and fluid overload before and the reasons for these elevated biomarkers remain incompletely understood [33, 34]. Some studies described levels of brain natriuretic peptides to be elevated in inflammation and cytokine activation [35, 36] which in the context of HR myeloma disease with an expected significant cytokine activation in the bone marrow microenvironment might well be a contributing factor.

Therefore, higher disease burden in NDMM patients compared to RRMM patients who undergo regular assessments of disease activity might be an indirect cause for higher NT-proBNP levels in our cohort via the above-mentioned mechanisms. Conversely, the reduction of NT-proBNP seen throughout Isa-KRd induction may be a result of disease control. Our finding that neither the baseline NT-proBNP value nor an early change from baseline within the first three induction cycles are predictive for CVAE are in contrast to prior results by Cornell et al. who showed that patients with elevated baseline BNP experienced CVAE more frequently. Importantly, their study was conducted in a RRMM setting where patients had most likely a lower disease burden compared to our cohort of NDMM patients. In addition, patients had been exposed to 1–9 prior treatment lines and more than half had received prior HDT with melphalan [16]. The cardiotoxicity may thus not be comparable to the here investigated newly diagnosed MM population receiving first line treatment. In fact, as more lines of prior treatment including anthracyclines are known to be a risk factor for cardiotoxicity [17, 29], conclusions drawn from heavily pretreated patient populations may not be generalizable to the newly diagnosed population. In line with our findings, a previous retrospective study described BNP increase from baseline during PI treatment of RRMM patients but did not find a correlation of biomarker increase and cardiovascular side effects [37]. Notably, our study showed higher baseline NT-proBNP levels in patients who experienced a cardiac dysfunction. However, the low event number (*n* = 10) prohibited further statistical analysis (e.g. time to event analyses, Cox regression analysis) to test the predictive power. In conclusion, elevated baseline NT-proBNP levels should be interpreted with caution and should not be used as a general reason to exclude carfilzomib-based treatment for disease control in patients with NDMM. However, larger studies are warranted to investigate whether NT-proBNP levels above a certain threshold yield predictive value for the occurrence of cardiac dysfunction.

Unlike NT-proBNP, we found hsTropI to be only rarely elevated at any timepoint, and values of ≥ 2.9 pg/mL (LOQ) as well as values greater to and equal to the median to be significantly associated with an increased risk for CVAE. The LOQ as the here identified threshold is rather low, indicating a higher risk already with hsTropI levels within the normal range. Larger studies are needed to validate the predictive value and determine optimal thresholds of hsTropI. However, while validation studies are pending, our data suggest that assessment of hsTropI at baseline may help identify patients at increased risk for CVAE who may profit from closer clinical monitoring for (even mild) symptoms and a rigorous management of cardiovascular risk factors.

Although this was a multi-center trial, our study has some important limitations, most importantly the limited patient number and consequently the small number of cardiovascular events limiting the statistical power of our analyses and the robustness of our conclusions. Statistical limitations due to the small number of events furthermore include the lack of adjustment for multiple comparisons and for demographics and clinical characteristics. In addition, the CONCEPT trial included exclusively high-risk patients, therefore it is not known whether findings differ in standard risk patients. However, as standard risk MM is generally associated with a lower disease burden than high risk MM [38–40], one might speculate that NT-proBNP levels might be less frequently elevated and therefore a more robust marker of cardiovascular risk in the standard risk setting. Another limitation of our study is that due to the quadruplet-regimen, potential interferences of the different study drugs cannot be differentiated and the findings of this study cannot be readily applied to all carfilzomib-based regimens. However, while the potential cardiovascular side effects of carfilzomib and lenalidomide are well-established [9, 12], there has been none such signal for isatuximab across any myeloma study to the best of our knowledge. Moreover, in line with study inclusion criteria, patients had overall only mild cardiovascular comorbidities and interference of unknown patient-specific cardiac risk factors (e.g., family history, physical activity etc.) cannot be ruled out.

In summary, our study suggests that elevated NT-proBNP levels are very common and not predictive of CVAE whereas low levels of hsTropI demonstrated a negative predictive value for the occurrence of CVAE in high-risk newly diagnosed MM patients in the CONCEPT trial undergoing carfilzomib-based Isa-KRd treatment. Our trial clearly adds to the knowledge base on risk assessment for cardiotoxicity in MM treatment, as it is one of the first to evaluate cardiac biomarkers in the first-line setting and is therefore hypothesis-generating for future studies. Larger studies primarily designed to prospectively assess CVAE are warranted to evaluate the predictive value and optimal cut-offs of cardiac biomarkers for cardiovascular risk in high-risk and standard-risk, newly-diagnosed and relapsed multiple myeloma patients. Ideally, adverse events should be assessed and classified by a cardiologist in real-time to increase the specificity of reported events. Meanwhile, elevated baseline NT-proBNP should not lead to withholding of carfilzomib-based treatment in the first-line setting and hsTropI at baseline may help to identify patients at increased risk for CVAE. In general, close monitoring optimally jointly with cardio-oncologists and adherence to guideline recommendations for optimizing cardiac risk factors should be undertaken for all patients to reduce the risk of cardiotoxicity [15, 18, 41].

## Supplementary Information

Supplementary Material 1.

## Data Availability

Requests for individual data should be directed to the corresponding authors; reasonable requests with profound methodological approach will be considered, and approval of the steering committee of the German-speaking Myeloma Multicenter Group must be obtained first.
