# Supplementary material for: Cardiac biomarkers for risk stratification in newly diagnosed high-risk multiple myeloma in the GMMG-CONCEPT trial
Source: Cardiooncology. 2025 Jul 3;11:63. doi: 10.1186/s40959-025-00358-x (PMC12224601; doi:10.1186/s40959-025-00358-x)
Supplement: Supplementary file 1 — Supplementary Material 1. [file 40959_2025_358_MOESM1_ESM.docx]

**Supplementary table 1: List of key inclusion/exclusion criteria and treatment information**

| **Parameter** | **Details** |
| --- | --- |
| **Key inclusion criteria** | Patients must satisfy the following criteria to be enrolled in the study:   1. Patients must have newly diagnosed, untreated, symptomatic (according to the revised C.R.A.B. criteria 2014), documented myeloma and have measurable disease (serum M protein ≥1 g/dL [for IgA ≥0.5 g/dL] or urine M protein ≥200 mg/24 hours); or, in case of oligosecretory myeloma, have involved FLC level ≥10 mg/dL, provided the sFLC ratio is abnormal; or, in case of non-secretory myeloma, have >1 focal lesion measurable by MRI.   Patients must have HR myeloma, defined as follows:   - Presence of ≥1 of the following cytogenetic abnormalities (determined by FISH): - del(17p) in ≥10% of purified cells - t(4;14) - >3 copies +1q21^a^ - t(14;16) - ISS stage II or III (all patients).  1. Must be ≥18 years of age at the time of signing the informed consent form. 2. Must be able to adhere to the study visit schedule and other protocol requirements in the investigator’s opinion 3. WHO performance status 0-3 (WHO = 3 is allowed only if caused by MM and not by comorbid conditions). 4. All patients have to adhere to a strict pregnancy prevention plan |
| **Key exclusion criteria** | The presence of any of the following will exclude a patient from enrollment:   1. Contraindication to any of the required concomitant drugs or supportive treatments 2. Patients with known systemic amyloidosis (except for AL amyloidosis of the skin or the bone marrow). 3. Administration of systemic chemotherapy, biological, immunotherapy, or any investigational agent (therapeutic or diagnostic) for MM, except bisphosphonate therapy. Emergency treatment with dexamethasone is allowed when the cumulative dexamethasone dose is ≤160 mg. It is allowed to include patients in the trial after 1 cycle (4 weeks) of any anti-myeloma first-line treatment. 4. Any of the following laboratory abnormalities:    - ANC <1,000/μL, unless related to myeloma    - Platelet count <30,000/μL (in case of platelets <50,000/µL and ≥30,000/µL myeloma bone marrow infiltration should be ≥50%)    - Corrected serum calcium >14 mg/dL (>3.5 mmol/L) or free ionized calcium >6.5 mg/dL (>1.6 mmol/L)    - Serum GOT/AST or SGPT/ALT >3.0 × ULN or serum total bilirubin >2.0 mg/dL if not due to hereditary abnormalities as Gilbert’s disease or hereditary hemolysis    - Patients with severe renal impairment (eGFR <30 mL/min/1.73 m², MDRD formula or CDK-EPI or creatinine clearance <30 mL/min) 5. Active congestive heart failure (NYHA Class III to IV), symptomatic cardiac ischemia, or conduction abnormalities uncontrolled by conventional intervention. Myocardial infarction within 4 months prior to study entry. 6. Known HIV seropositive, hepatitis C infection, and/or hepatitis B 7. Acute active, uncontrolled infection. 8. Significant neuropathy (grades 3 to 4 or grade 2 with pain according CTCAE V4.03) 9. Second malignancy within the past 5 years, except:   - Adequately treated basal cell or squamous cell skin cancer  - Carcinoma in situ of the cervix  - Prostate cancer Gleason Score ≤6 with stable PSA over the past 12 months  - Breast carcinoma in situ with full surgical resection  - Treated medullary or papillary thyroid cancer.   1. Patients with pleural effusions requiring thoracentesis or ascites requiring paracentesis within 14 days prior to study entry. 2. Major surgery within 4 weeks prior to cycle 1, day 1 (kyphoplasty is not considered major surgery); patients should have been fully recovered from any surgical-related toxicities. 3. Any other clinically significant medical disease or psychiatric condition that, in the investigator’s opinion, may interfere with protocol adherence or a patient’s ability to give informed consent. |
| **Treatment schedule**  **Induction and consolidation** | Cycle length: 28-day period  Isatuximab: 10 mg/kg intravenously (IV) on days 1, 8, 15, and 22 during the initial cycle and on days 1 and 15 in subsequent cycles.  Carfilzomib: 20 mg/m^2^ IV on days 1 and 2, and 36 mg/m^2^ IV on days 8, 9, 15, and 16 during the first cycle, followed by 36 mg/m^2^ IV on days 1, 2, 8, 9, 15, and 16 in subsequent cycles (27 mg/m^2^ throughout consolidation cycle 1).  Lenalidomide: 25 mg orally on days 1 through 21 (15 mg throughout consolidation cycle 1)  Dexamethasone: 40 mg orally or IV on days 1, 8, 15, and 22 (20 mg for patients aged >75 years).  Thromboembolic prophylaxis was mandatory throughout the trial. |
| **Treatment schedule maintenance** | Cycle length: 28-day period  Isatuximab: 10 mg/kg intravenously (IV) on days 1 and 15  Carfilzomib: 70 mg/m^2^ IV on days 1 and 15  Lenalidomide: 15 mg orally on days 1 through 21  Thromboembolic prophylaxis was mandatory throughout the trial. |

AL, amyloid light chain; ALT, alanine aminotransferase; ANC, absolute neutrophil count; AST, aspartate aminotransferase; CDK-EPI, chronic kidney disease epidemiology collaboration; C.R.A.B., calcium elevation, renal insufficiency, anemia, and bone abnormalities; CTCAE, common terminology criteria for adverse events; eGFR, estimated glomerular infiltration rate; FISH, fluorescence in situ hybridization; FLC, free light chain; GOT, glutamic oxaloacetic transaminase; HR, high-risk; IgA, immunoglobulin A; ISS, International Staging System; MDRD, modification of diet in renal disease; MM, multiple myeloma; MRI, magnetic resonance imaging; NYHA, New York Heart Association; PSA, prostate-specific antigen; sFLC, serum free light chain; SGPT, glutamic-pyruvic transaminase; ULN, upper limit of normal; WHO, World Health Organisation.

^a^Following a protocol amendment in 2021, patients with three copies of 1q21 could also be included in the second cohort from 2021 onward.

**Supplementary table 2: List of “other cardiovascular comorbidities (CVM)”**

| **Other CVM** | **Frequency (n)** |
| --- | --- |
| Mitral valve regurgitation | 6 |
| Aortic valve regurgitation | 1 |
| Aortic valve stenosis | 1 |
| Tricuspid valve regurgitation | 5 |
| Pulmonary valve regurgitation | 1 |
| Cardiomegaly | 1 |
| Hypermobility of interatrial septum | 1 |
| Left ventricular hypertrophy | 2 |
| Aorta ascendens ectasia | 1 |
| Status post aortic aneurysm stent implantation | 1 |
| Status post parainfectious myocarditis | 1 |
| Status post transient ischemic attack (TIA) | 2 |

CVM, cardiovascular comorbidities.

All severity grades (e.g., mild, moderate) for each CVM were summarized.

**Supplementary Table 3: NT-proBNP levels in pg/mL depending on prevalence of CVRF**

| **Time point** | **CVRF** | | |
| --- | --- | --- | --- |
|  | **absent**  **n=77** | **present**  **n=49** | **p-value** |
| **T1** | 194 (129, 469) | 329 (138, 646) | 0.17 |
| **T2** | 134 (71, 248) | 215 (74, 803) | 0.26 |
| **T3** | 152 (65, 306) | 147 (76, 215) | 0.76 |
| **T4** | 106 (64, 155) | 117 (77, 309) | 0.26 |
| **T5** | 112 (62, 226) | 167 (87, 580) | 0.059 |
| **T6** | 167 (8, 260) | 142 (89, 291) | 0.70 |

CVRF, cardiovascular risk factors.

Medians (interquartile range) are displayed. Kruskal-Wallis test was applied, a p-value < 0.05 was considered significant.

**Supplementary Table 4: hsTropI levels in ng/L depending on prevalence of CVRF**

| **Time point** | **CVRF** | | |
| --- | --- | --- | --- |
|  | **absent**  **n=77** | **present**  **n=49** | **p-value** |
| **T1** | 3.6 (2.2, 5.7) | 3.8 (2.7, 9.5) | 0.28 |
| **T2** | 3.2 (2.3, 5.0) | 3.5 (2.3, 7.4) | 0.30 |
| **T3** | 2.7 (2.2, 5.0) | 3.8 (2.4, 4.5) | 0.46 |
| **T4** | 3.2 (2.0, 4.6) | 4.0 (2.9, 6.7) | 0.16 |
| **T5** | 3.3 (2.0, 5.6) | 3.1 (2.3, 6.6) | 0.75 |
| **T6** | 4.5 (2.7, 7.0) | 3.8 (2.2, 8.2) | 0.44 |

CVRF, cardiovascular risk factors.

Medians (interquartile range) are displayed. Kruskal-Wallis test was applied, a p-value < 0.05 was considered significant.

**Supplementary Table 5: NT-proBNP levels in pg/ml depending on a stricter CVAE definition**

| **Time point** | **CVAE_2_**  **(excluding thromboembolic events and stroke)** | | |
| --- | --- | --- | --- |
|  | **absent**  **n=96** | **present**  **n=30** | **p-value** |
| **T1** | 212 (118, 510) | 257 (168, 496) | 0.32 |
| **T2** | 158 (87, 366) | 138 (64, 858) | 0.82 |
| **T3** | 148 (76, 274) | 159 (63, 319) | 0.77 |
| **T4** | 97 (65, 187) | 118 (74, 309) | 0.22 |
| **T5** | 146 (59, 279) | 154 (91, 382) | 0.15 |
| **T6** | 135 (63, 255) | 224 (155, 427) | **0.0032** |

CVAE, cardiovascular adverse events.

Medians (interquartile range) are displayed. Kruskal-Wallis test was applied, bold p-value indicates significance (p-value < 0.05).

**Supplementary Table 6: NT-proBNP levels in pg/ml depending on occurrence of cardiac dysfunction**

| **Time point** | **Cardiac dysfunction** | | |
| --- | --- | --- | --- |
|  | **absent**  **n=116** | **present**  **n=10** | **p-value** |
| **T1** | 212 (127, 480) | 460 (220, 1072) | 0.06 |
| **T2** | 148 (71, 366) | 225 (127, 2176) | 0.33 |
| **T3** | 148 (75, 295) | 147 (65, 530) | 0.82 |
| **T4** | 112 (72, 206) | 63 (51, 147) | 0.61 |
| **T5** | 145 (64, 285) | 171 (109, 688) | 0.16 |
| **T6** | 153 (85, 259) | 260 (205, 996) | **0.028** |

Cardiac dysfunction is defined as incident heart failure, LVEF reduction and diastolic dysfunction of any grade.

Medians (interquartile range) are displayed. Kruskal-Wallis test was applied, bold p-value indicates significance (p-value < 0.05).

**Supplementary Table 7: hsTropI levels in ng/L depending on a stricter CVAE definition**

| **Time point** | **CVAE_2_**  **(excluding thromboembolic events and stroke)** | | |
| --- | --- | --- | --- |
|  | **absent**  **n=96** | **present**  **n=30** | **p-value** |
| **T1** | 3.6 (2.1, 6.9) | 4.0 (3.0, 7.4) | 0.16 |
| **T2** | 3.2 (2.3, 4.7) | 6.8 (2.3, 10.1) | **0.031** |
| **T3** | 2.8 (1.8, 4.2) | 4.3 (2.7, 5.5) | **0.033** |
| **T4** | 3.2 (2.0, 5.8) | 3.4 (2.9, 4.2) | 1.00 |
| **T5** | 3.1 (2.0, 6.0) | 3.7 (2.7, 6.2) | 0.39 |
| **T6** | 3.5 (2.2, 5.9) | 7.0 (5.4, 8.7) | **<0.001** |

CVAE, cardiovascular adverse events.

Medians (interquartile range) are displayed. Kruskal-Wallis test was applied, bold p-value indicates significance (p-value < 0.05).

**Supplementary Table 8: hsTropI levels in ng/L depending on cardiac dysfunction**

| **Time point** | **Cardiac dysfunction** | | |
| --- | --- | --- | --- |
|  | **absent**  **n=116** | **present**  **n=10** | **p-value** |
| **T1** | 3.7 (2.2, 6.8) | 4.2 (3.7, 14.8) | 0.16 |
| **T2** | 3.5 (2.3, 5.2) | 5.5 (2.0, 10.1) | 0.48 |
| **T3** | 2.9 (2.0, 4.5) | 3.0 (2.2, 4.9) | **0.039** |
| **T4** | 3.2 (2.0, 5.1) | 4.0 (3.1, 4.1) | 0.81 |
| **T5** | 3.2 (2.2, 6.1) | 3.3 (2.4, 6.2) | 0.73 |
| **T6** | 3.8 (2.5, 7.0) | 8.0 (5.2, 8.8) | 0.090 |

Cardiac dysfunction includes incident heart failure, LVEF reduction and diastolic dysfunction of any grade.

Medians (interquartile range) are displayed. Kruskal-Wallis test was applied, bold p-value indicates significance (p-value < 0.05).

**Figure Legends**

**
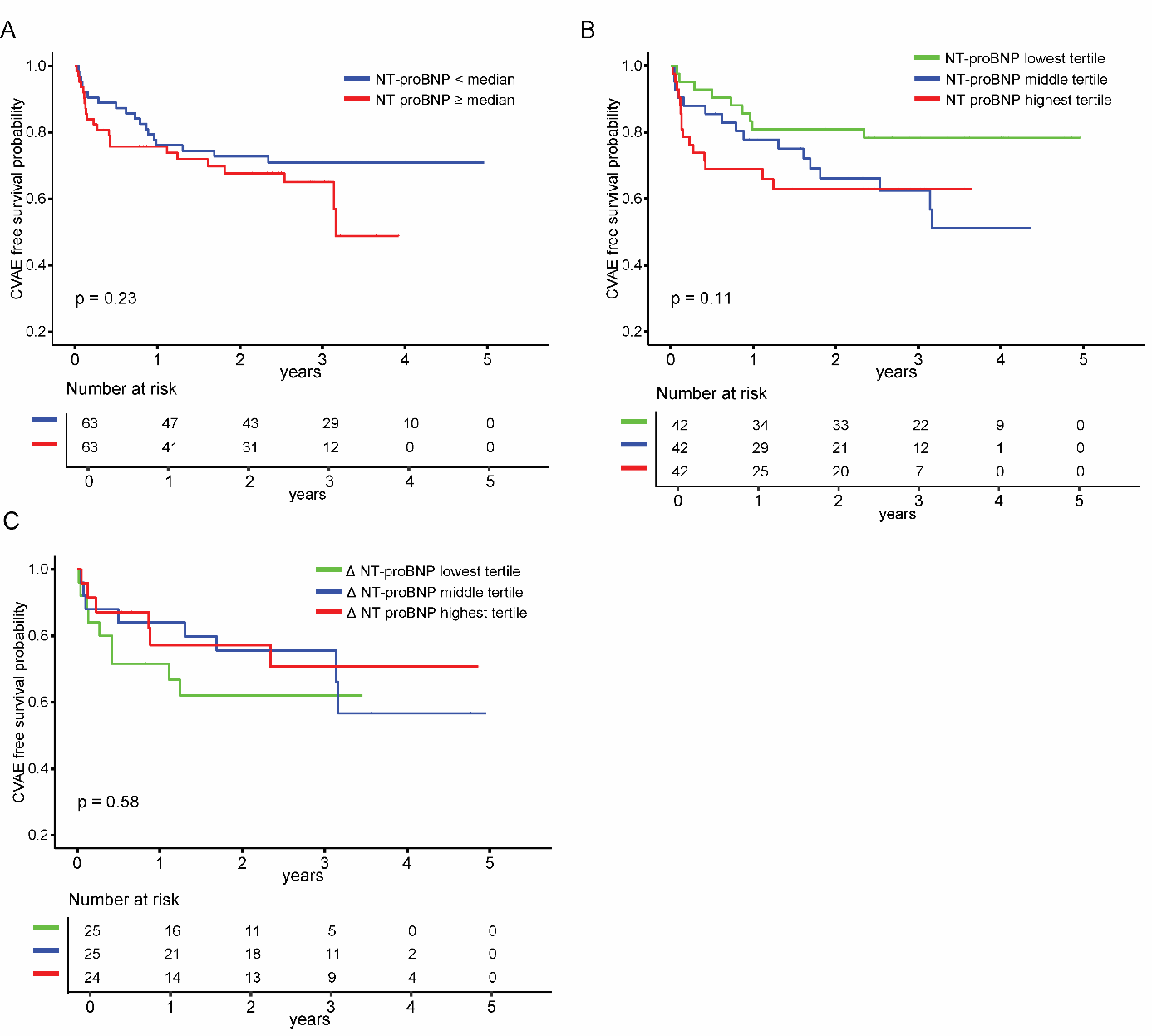
Supplementary Figure 1 CVAE free survival depending on NT-proBNP**

NT-proBNP levels were measured using immuno-assays in patients before initiation (n=126, T1) and during the first three cycles (n=74, T2) of carfilzomib-based quadruplet therapy for newly diagnosed high risk multiple myeloma within the GMMG-CONCEPT study. Occurrence of cardiovascular adverse events was assessed via electronic case report forms. Time-to-event analysis was performed stratifying patients by (A) NT-proBNP levels below the median and greater to and equal to the median, (B) by NT-proBNP tertiles, and (C) ΔNT-proBNP (NT-proBNP_T2_ - NT-proBNP_T1_) tertiles. Log-rank test was used for statistical analysis. P-values < 0.05 were considered significant.

**
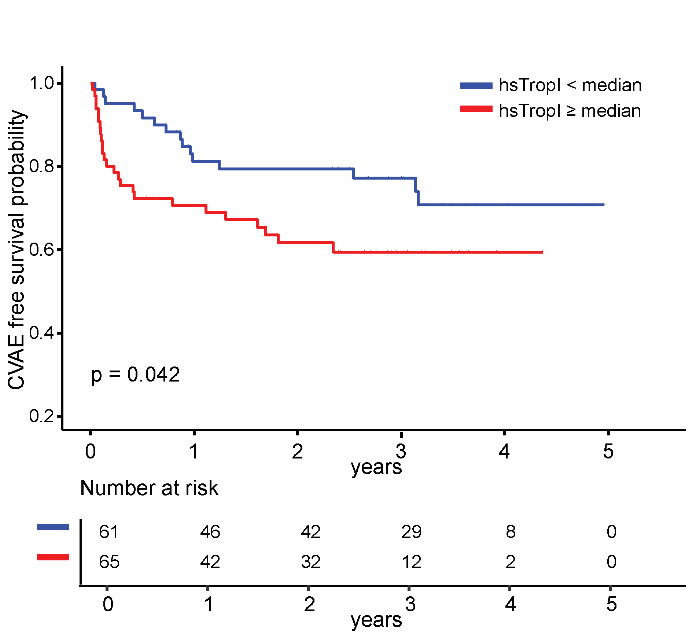
Supplementary Figure 2 CVAE free survival depending on hsTropI levels**

High sensitive troponin I (hsTropI) levels were measured using immuno-assays in patients before initiation (n=126, T1) and during the first three cycles (n=74, T2) of carfilzomib-based quadruplet therapy for newly diagnosed high risk multiple myeloma within the GMMG-CONCEPT study. Occurrence of cardiovascular adverse events was assessed via electronic case report forms. Time-to-event analysis was performed stratifying patients by hsTropI below the median and greater to and equal to the median. Log-rank test was used for statistical analysis. P-values < 0.05 were considered significant.
